# Clinical and Pathological Features of Osteosarcomas of the Jaws: A Retrospective Study

**DOI:** 10.3390/clinpract14030077

**Published:** 2024-05-23

**Authors:** Jesus Rodriguez-Molinero, Jose Juan Pozo-Kreilinger, Juan Antonio Ruiz-Roca, Antonio Francisco Lopez-Sanchez, Jose Luis Cebrian-Carretero

**Affiliations:** 1 Department of Nursery and Stomatology, Faculty of Health Sciences, Rey Juan Carlos University, 28922 Alcorcón, Madrid, Spain; antonio.lopez@urjc.es; 2 High-Performance Research, Development and Innovation Group in Dental Biomaterials of Rey Juan Carlos University, 28922 Alcorcón, Madrid, Spain; 3 Department of Pathology, Hospital Universitario La Paz, 28046 Madrid, Spain; 4 Department of Dermatology, Stomatology and Radiology, Faculty of Dentistry, University of Murcia, 30100 Murcia, Spain; jaruizroca@um.es; 5 Oral and Maxillofacial Surgery Department, University Hospital La Paz, Paseo de la Castellana, 261, 28046 Madrid, Spain

**Keywords:** osteosarcoma of the jaw, head and neck cancer, oral cancer, malignant bone tumors

## Abstract

Introduction: Osteosarcomas of the jaw (OSJs) are rare tumors with distinct characteristics from osteosarcomas affecting other bones. This study aims to analyze the clinical, pathological, and therapeutic characteristics of OSJs. Methods: A retrospective, descriptive cross-sectional study including patients diagnosed with OSJ registered at the “La Paz” University Hospital, Madrid, was performed. Results: Data of eight patients with a diagnosis of OSJ were obtained during the study period of 22 years (2002–2024). The mean age of the patients was 41 years. The distribution was 1:1 between the maxilla and mandible. Painful inflammation was the most frequent clinical manifestation. Conventional osteoblastic osteosarcoma was the most predominant histological type. Survival rate at 5 years was 50%, which decreased to 25% at 10 years. Conclusions: OSJs differ from conventional osteosarcomas of long tubular bones. Surgery continues to be the mainstay of treatment. However, more studies are needed through which more standardized protocols can be proposed for adjuvant therapeutic management.

## 1. Introduction

Osteosarcoma (OS) is a primary malignant tumor lesion of mesenchymal origin that shows osteogenic differentiation [1]. Typically affecting long bones (femur, tibia, and humerus) [2], osteosarcomas within the maxillofacial region are less common, comprising approximately 6–7% of all OSs [3]. Osteosarcomas of the jaw (OSJs) represent 6% of tumors affecting the jaw and less than 1% of all malignant head and neck tumors [4]. Unlike other osteosarcomas that are more common in children and adolescents, OSJs affect individuals in their third and fourth decades of life [5]. The mandible is the most affected bone in this region [6], with no notable sex predilection [7].

The etiopathogenesis of OSJ remains uncertain, although chromosomal events that give rise to heterogeneous and complex chromosomal aberrations must be involved [8].

Osteosarcomas typically manifest as central or intramedullary lesions, with conventional OS standing out as the most prevalent subtype. This type of OS, in turn, can be classified into three subtypes according to its predominant cell differentiation and production of extracellular matrix: osteoblastic, chondroblastic, or fibroblastic, although they can present as a mixed formation [9,10]. Other more unusual variants of OS, such as telangiectatic, small cell, epithelioid, and multinucleated giant cell-rich, have also been reported [9]. In addition to central lesions, OSs can appear on a bony surface or as juxtacortical lesions (subdivided further into parosteal OS, periosteal OS, or surface OS) and as extraskeletal or soft-tissue OS [6].

The clinical symptoms of OSJ vary, with inflammation, paresthesia, and pain commonly observed in 3–8% of cases [11]. Other associated signs are tooth displacement or loss and misalignment of the removable dental prosthesis [11].

OS can have a primary or secondary origin, with predisposing factors related to certain pathologies, such as Paget’s disease, Li–Fraumeni syndrome, Rothmund–Thompson syndrome type 2, Werner syndrome, Rapadilino syndrome, Bloom syndrome, and intraosseous diseases including fibrous dysplasia, ossifying fibroma, and hereditary retinoblastoma. Moreover, OS can also originate in patients with prior radiotherapy treatment in the head and neck region [12,13,14].

The mainstay of treatment for OSJ is surgical resection of the tumor. Tumor infiltration-free margins are a prognostic factor for survival [15]. Other strategies include the administration of neoadjuvant chemotherapy or adjuvant therapy with chemotherapy or radiotherapy [16], although no established protocol exists due to the low prevalence rate of this tumor lesion.

The prognosis of OSJ is better than that of OS affecting extremities, with better survival data and fewer distant metastases [17,18].

The objective of this study is to review the cases of osteosarcomas of the jaw diagnosed and treated in the Oral and Maxillofacial Surgery Service of the La Paz University Hospital between 2002 and 2024. This study sought to explore the clinicopathological, therapeutic, and prognostic characteristics, analyzing survival in this type of tumor and comparing findings with other cases published in the scientific literature.

## 2. Material and Methods

### 2.1. Study Design

To conduct this retrospective, descriptive cross-sectional study, a search was carried out in the database of the Oral and Maxillofacial Surgery Service and Pathological Anatomy Service of the University Hospital La Paz in Madrid, Spain, focusing on cases diagnosed as OSJ in the last 22 years.

### 2.2. Participant Selection Criteria

Patient selection was meticulously conducted through a comprehensive review of clinical records housed within the repository of the Oral and Maxillofacial Surgery Service. In order to ensure data integrity and prevent any potential loss, cross-referencing was performed between this repository and the database housed within the Pathology Department of Hospital La Paz.

Inclusion criteria encompassed individuals of all ages and genders who had received an initial diagnosis and subsequent treatment within the confines of the hospital premises. Patients failing to meet these predetermined inclusion criteria were subsequently excluded from the study cohort.

In each case under investigation, a standardized protocol was adhered to, which entailed the performance of an initial biopsy to validate the diagnostic suspicion of osteosarcoma of the jaw (OSJ) in conjunction with a computed tomography (CT) scan. This rigorous approach ensured the confirmation of diagnoses through histopathological examination while concurrently providing detailed radiographic imaging to further delineate the extent and characteristics of the osseous lesion. Subsequently, the studies were complemented with a PET–CT scan (positron emission tomography–computed tomography) to rule out metastatic focus during diagnosis.

Data were collected regarding the clinical characteristics, location of the affected bone, type and quality of surgical treatment, administration of neoadjuvant and/or adjuvant treatment, pathological characteristics of the lesions, soft-tissue invasion, recurrences and metastases, and 5-year survival outcomes. We meticulously recorded all cases to avoid potential bias, capturing data about the characteristics mentioned earlier. When this information was absent from the clinical records, we noted it in the registry table as “no data”.

### 2.3. Calculation of Participant Survival

The Kaplan–Meier method [19], a widely employed nonparametric approach in survival analysis, was utilized to compute the cumulative probability of patient survival. This method is particularly advantageous because it accounts for censored observations, enabling the estimation of survival probabilities over time, even in the presence of incomplete follow-up data. This study used the statistical software GraphPad Prism 9.0 (GraphPad Software, La Jolla, CA, USA) to calculate data.

### 2.4. Ethical Aspects

Ethical approval for this study was obtained from the ethics committee of the Hospital La Paz IdiPAZ Research Institute (HULP: PI-5462), and the study adhered to the principles outlined in the Declaration of Helsinki [20].

## 3. Results

### 3.1. Characteristics of the Patients

A total of eight patients with OSJ were registered in the last 22 years. The age of the diagnosed patients was between 10 and 56 years, with a mean age of 41 ± 14.75 SD (standard deviation) years. Notably, 87.5% (*n* = 7) of the patients were women.

Regarding the origin of the OS tumors, the cohort comprised five cases of primary OS and three cases of secondary OS. Among the secondary OS cases, one was induced by radiation following the treatment of cavum cancer with chemotherapy and radiotherapy 11 years earlier, the second developed subsequent to an ossifying fibroma in the same area 5 years earlier, and the third was associated with chemotherapy (four cycles of etoposide and carboplatin) and tomotherapy following retinoblastoma in a pediatric patient.

**Figure 1:**
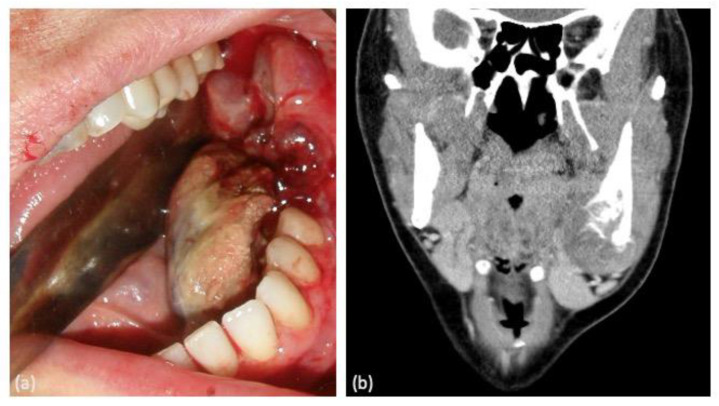
(**a**) Intraoral clinical manifestation of the tumor in the left mandibular molar area showing bleeding. (**b**) Computed tomography coronal section image of the same lesion showing an area of osteolysis.

In terms of clinical manifestations of the tumor, painful inflammation was the most prevalent symptom (62.5%), either as a primary symptom or in association with other symptoms such as bleeding, dental mobility, and hypoesthesia of the inferior alveolar nerve. Notably, two cases presented with asymptomatic inflammation causing facial deformity (Figure 1).

With respect to the affected bone, an equal distribution (1:1 ratio) was observed between the maxilla and mandible, as well as between the sides where the lesion was located. A 50% distribution was found for both the right and left sides (Table 1).

### 3.2. Pathological Characteristics of Tumor Lesions

**Figure 2:**
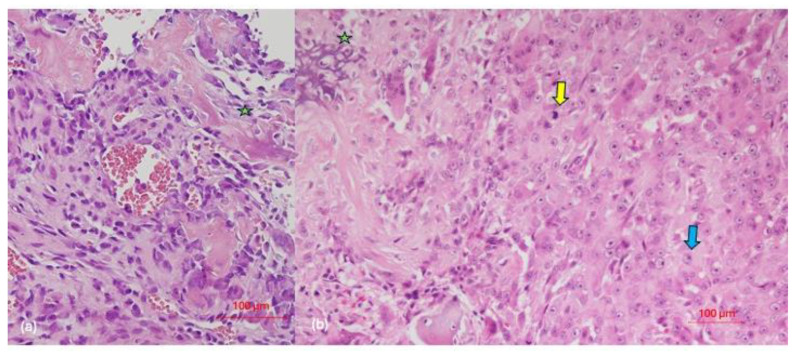
(**a**) Conventional osteoblastic osteosarcoma (hematoxylin and eosin [HE] ×400): a malignant mesenchymal cell proliferation with cytologic atypia, characterized by variability in cell shape and size, along with anisokaryosis. Nucleoli are evident. The neoplastic cellularity varies in shape from polygonal to fusiform, with focal observation of calcified collagenous matrix production (osteoid). (**b**) Epithelioid osteosarcoma (HE ×400): hypercellular malignant mesenchymal proliferation, characterized by polygonal tumor cells with abundant cytoplasm and nucleus with open chromatin and prominent nucleolus. The osteoid matrix is associated with neoplastic cellularity. Green star: osteoid formation by malignant tumor cells; yellow arrow: cell mitosis; blue arrow: epithelioid tumor osteoblast.

All registered cases exhibited high-grade OSJ with a predominance of osteoblastic morphological pattern (n = 3), followed by chondroblastic (n = 2), epithelioid (n = 2), and fibroblastic (n = 1) patterns. According to the Broder’s classification [21], grade 4 was the most prevalent (two cases of osteoblastic OSJ and one case of epithelioid OSJ), along with grade 3 (two cases of osteoblastic OSJ and one chondroblastic). In addition, cases of grade 3–4 fibroblastic OSJ and a case of grade 2–3 chondroblastic OSJ were noted (Figure 2).

In 50% of the cases, soft tissues were infiltrated; particularly during its recurrence in case 2.

Lymphovascular invasion was detected in two cases (cases 2 and 8). In case 2, it occurred following tumor recurrence (Table 2).

### 3.3. Treatment Characteristics and Results

**Figure 3:**
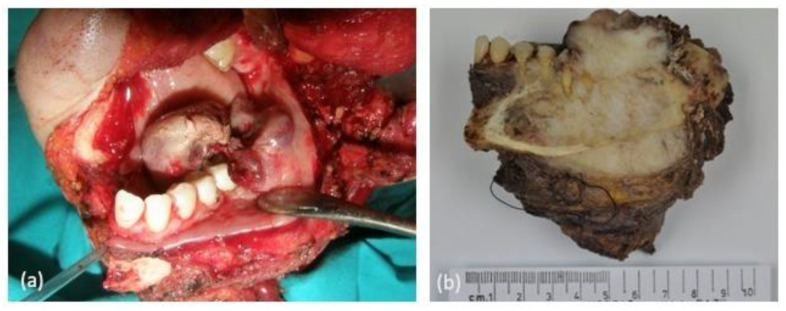
(**a**) Left hemimandibulectomy with safety margins. (**b**) Surgical piece for anatomopathological study.

The surgical treatment of choice in all cases was resection of the tumor by partial maxillectomy or hemimandibulectomy (Figure 3).

Complete resection with the presence of tumor-free margins and no residual tumor (R0) was identified in six cases, of which three were mandibular OSJ and three were maxillary OSJ. Notably, in case 2, affected edges were found following the second local recurrence.

Neoadjuvant therapy was administered in three cases. In two cases, the same protocol was used, with cisplatin and adriamycin administered for only one cycle due to tumor progression. Assessing the percentage of necrosis was challenging in the histological study of the surgical specimen. The third case, being a pediatric patient with maxillary OSJ, was administered neoadjuvant therapy using the ISG-GEIS-OS2 protocol (Italian Sarcoma Group–Spanish Group for Research on Sarcomas–Osteosarcomas 2) involving the administration of methotrexate, adriamycin, and cisplatin. In this case, 95% tumor necrosis was observed.

Adjuvant therapy was administered in five cases initially and in another two cases after tumor recurrence (cases 2 and 3). In only one case, adjuvant therapy was not administered due to the presence of free tumor margins after surgical treatment and the absence of lymphovascular or soft-tissue invasion (case 4). The most commonly used protocol was that involving cisplatin–adriamycin for five to six cycles. In two cases of epithelioid OSJ, one cycle of the second-line chemotherapy with ifosfamide or ifosfamide-etoposide was used (cases 3 and 8) due to the recurrence, progression, and aggressiveness of the tumor.

All chemotherapy regimens were completed except in case 8, in which neoadjuvant chemotherapy had to be discontinued due to tumor progression. Additionally, adjuvant chemotherapy was ineffective in the same case.

Radiotherapy (tomotherapy) was administered along with chemotherapy (ifosfamide–cisplatin) in only one case and for five cycles (case 1). Local recurrence was the most common complication, observed in three cases (two in the mandible (cases 3 and 8) and one in the maxilla (case 2)). The epithelioid type was the most prevalent histological subtype, followed by the chondroblastic type. Specifically, in the case of maxillary OSJ with a chondroblastic pattern, three local recurrences of the same tumor were noted (case 2). Distant metastases were detected in only one case, involving the lung and liver.

In this survival study, only two cases of death were recorded; both occurred within 1 year from diagnosis. Two cases of epithelioid OSJ accounted for 0.58 years lapsed between the diagnosis of osteosarcoma and the end of the study, corresponding to the two patients with epithelioid OSJ who died during this period, while the other patients had been in the study for 15.83 years, corresponding to a case of OSJ of the osteoblastic type and another of the fibroblastic type. The mean follow-up time of the patients was 6.5 years. (Table 3).

**Figure 4:**
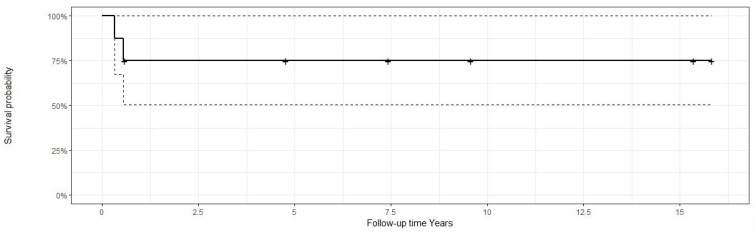
Kaplan–Meier survival diagram for osteosarcoma.

Therefore, it can be established that the survival rate was 85% within the first year, decreasing to 75% at 1 year and remaining constant in the subsequent years. (Figure 4).

## 4. Discussion

Osteosarcoma of the jaw and osteosarcoma of the extremities share morphological characteristics, although OSJ differs in several aspects. The differences in age distribution are evident, as OS of the extremities tends to affect younger patients [22]. A significant contrast lies in the clinical realm, particularly regarding the emergence of metastatic dissemination, which is the primary determinant of patient prognosis. Hematogenous dissemination is less frequent (6–21% of patients) and tends to occur in more advanced stages of the disease [23]. In OS of the extremities, the possibility of primary metastasis exists in 25% of cases, with pulmonary involvement being the most common [24]. One of the main differences between these osteosarcomas is their response to chemotherapy. It has been observed that extremity osteosarcomas usually respond better to neoadjuvant chemotherapy, which may facilitate subsequent surgical resection and improve long-term outcomes. In contrast, OSJ’s response to chemotherapy may be less predictable and require more individualized therapeutic approaches [23]. 

OSJ is a rare entity characterized by a series of clinicopathological features that differentiate it from OS of the extremities. During the last 22 years, eight cases of OSJ have been registered at the La Paz University Hospital. Large case series on OSJ are relatively scarce, with most studies documenting 8–28 cases over 10–30 years [10,25,26,27,28,29,30,31,32]. The most extensive case series included 74–114 cases, although these studies spanned much longer study periods [33,34]. Notably, a recent study by Brown et al. [35] in which 164 cases are recorded is the most extensive case series in the literature. Although in our study, the age range at diagnosis was wide (from 10 to 56 years), with a mean age of 41 years, in general, OSJ occurs in older patients: Typically, it is diagnosed between the third and fifth decade of life, with a mean age of between 34 and 36 years. These findings are consistent with those published in the scientific literature [10,27,29,32,34,36,37,38].

Most studies suggest an equal distribution of OSJ between the maxilla and mandible, although with a slight predilection for the latter [36]. When considering the age of lesion onset with respect to the affected bone, Paparella et al. [33] observed a greater peak incidence in the maxilla in the fifth decade of life compared to a more even distribution between young and older patients in OSJ involving the mandible. Garrington et al. [36] attributed this observation to the presence of growth centers in the mandible that allow for potential growth activity throughout life.

The distribution in terms of sex remains clear and is a subject of controversy [10]. Mardinger et al. [30] suggested a slight predilection for the male sex; however, in our study, we observed the opposite trend, with a clear predominance of OSJ in women (87.5% of cases), although it is true that our sample size was relatively small.

The most frequent clinical manifestation of OSJ is local inflammation, often accompanied by pain [32,39], as corroborated by our study. However, other symptoms can be observed, such as mobility or tooth loss and hypoesthesia of the dental nerve or visual disturbances such as proptosis or diplopia [28]. Radiologically, diverse findings may be present. The appearance of a “sunburst pattern” has often been pathognomonic of OSJ; however, it is not exclusive to this type of lesion, and it is not the most common radiological manifestation [33]. Several researchers in larger case series have observed the presence of mixed radiological images, which requires a thorough differential diagnosis [33,36,40,41].

Histologically, for a definitive OS diagnosis, a malignant mesenchymal tumor, along with the production of bone or osteoid matrix, must be present [42]. Controversy exists regarding the higher prevalence of one histological type over another, since some authors describe the chondroblastic type as the most predominant subtype in OSJ cases [27,30,32]. However, in our study, the osteoblastic type was the most common subtype, consistent with reports by other authors [10,32,33,36,38]. Regarding fibroblastic differentiation, there is a greater consensus regarding its lower incidence in OSJ [25,27]. OS of the epithelioid type in the jaw region is a rare variant, with only six cases published [7,43,44,45,46,47]. This variant, which was initially described by Scranton et al. [48] in 1975, is more prevalent in long bones and in men (2:1), presents between the first and seventh decade of life, and has a predilection for the mandible. It is radiologically identified as a poorly defined lytic lesion with the ability to show a periosteal reaction [43]. The presence of osteoid is a fundamental requirement for diagnosing OSJ, although the amount present can be variable [7]. In our study, two cases were recorded with this morphological pattern in which an extensive neoformation of osteoid in the socket was identified in close contact with the tumor cellularity. These cases corresponded to two women aged 56 and 31 years who presented OSJ with an epithelioid origin in the mandible and maxilla, respectively, and died within a period of less than 7 months. This aggressive behavior has already been described by other authors, such as Okada et al. [44] However, more studies are needed to analyze the clinical behavior of this histological variant in a more extensive way.

Special attention should be paid to cases of OS that are diagnosed with a history of radiotherapy to the head and neck [32]. This type of OS is generally of a high grade and associated with a worse prognosis [45]. In the case recorded in our study, this tumor type was confirmed as a high-grade and more aggressive lesion.

Certain conditions elevate the risk of developing OSJ. Patients with Li–Fraumeni syndrome or hereditary retinoblastoma exhibit chromosomal alterations in p53 and the retinoblastoma genes located at 17p13 and 13q14 that increase this risk [46]. OS can also develop in patients with Paget’s disease. This is a quite rare condition since it usually affects only 1% of patients, with a greater predominance in the long bones [47].

Other predisposing factors, such as cemento-ossifying fibroma, must also be considered [7]. In our study, we observed the presence of an initial lesion diagnosed as a cemento-ossifying fibroma that after 5 years transformed into chondroblastic OSJ. Therefore, accurate diagnosis is crucial since OS can be confused with benign fibro-osseous lesions, such as fibrous dysplasia [38], and other tumors, such as osteoblastomas [49].

The treatment protocol for OSJ is not standardized and varies greatly between institutions [50]. However, surgical treatment, including radical resection with wide free margins, continues to be the main option with the best prognosis for this type of tumor [26,28,30,51]. Achieving free margins is considered technically more difficult in the maxilla than in the mandible, with data ranging between 30% and 52% of affected margins after surgery [15,29,52]. In our study, among four patients with OSJ involving the maxilla, free margins were achieved in three; however, one of them experienced local recurrence, leading to affected margins.

Neoadjuvant (neoCTX) and adjuvant chemotherapy (CTX) are currently considered an essential complement to surgical treatment, especially in the management of high-grade osteosarcoma, particularly in long bones [53].

The NCCN Guidelines for Osteosarcoma outline a comprehensive treatment approach for high-grade osteosarcoma, encompassing intramedullary and surface presentations. The management strategy begins with neoadjuvant treatment, consisting of preoperative chemotherapy. Following this, restaging is conducted using various imaging modalities, including chest CT, contrast-enhanced MRI, and FDG-PET/CT or bone scan. Adjuvant treatment options are then determined based on the resectability of the tumor and response to preoperative chemotherapy. For unresectable tumors, radiotherapy and chemotherapy are recommended. For resectable tumors with positive margins, additional local therapy (surgical resection ± radiotherapy) is considered, with chemotherapy for cases showing a good response and potential modification of chemotherapy for cases with a poor response. For tumors with negative margins, chemotherapy is administered based on response to preoperative chemotherapy [54].

However, the management of OS of the head and neck is not standardized and varies greatly between institutions [50,55]. There are no established protocols regarding the use of CTX, given the rarity of this type of lesion and the lack of longitudinal data [56].

It is worth mentioning studies such as that by Woll et al., which investigated the use of doxorubicin, ifosfamide, and lenograstim as adjuvant chemotherapy treatment in other types of sarcomas, such as soft-tissue sarcomas [57]. In addition, the treatment of OS of the head and neck has been guided mainly by the treatment of long bones [58]. Since the introduction of adjuvant CTX in the treatment of long-bone OS, the 5-year survival rate of these patients has increased considerably from 20% in the 1970s to 50%. This increase is even more evident, at up to 70%, with the addition of cisplatin and ifosfamide to doxorubicin and methotrexate [59].

In contrast, the use of neoCTX has improved survival in patients with OS of long bones, but its application in OSJ remains controversial. Some researchers contend that neoadjuvant chemotherapy reduces tumor size, facilitating the attainment of negative margins during surgery [60]. Additionally, it affords surgeons ample preoperative time for surgical planning and enables immediate therapy initiation, circumventing the need for delayed surgery. In a retrospective analysis involving 201 patients, Smeele et al. underscored a notable enhancement in survival rates among patients treated with chemotherapy [61]. These findings suggest that neoadjuvant and adjuvant chemotherapy may elevate disease-free survival probabilities from 10–20% to 60% [58].

A recent systematic review by Khadembaschi et al. [62] reported no survival benefit of neoCTX compared to surgery as the primary treatment modality in the treatment of head and neck OSs. Tumor necrosis ranged from 0% to 76% (note that necrosis is effective when it is greater than 90% [62]). Even worse survival outcomes may be observed when surgical treatment is delayed [63,64]. Nevertheless, limited data elucidate the mechanism of chemoresistance in osteosarcoma, with some researchers suggesting alterations in DNA repair mechanisms, drug inactivation, or alterations in the cell cycle [65,66,67]. In our study, the effectiveness of this type of treatment was observed in a single case—specifically, a pediatric patient—with neoCTX yielding a tumor necrosis of 95%. However, due to limitations in the number of cases analyzed, we cannot really establish whether neoadjuvant chemotherapy is beneficial in all cases.

It is important to note that patients undergoing chemotherapy for cancers such as osteosarcoma may experience a variety of side effects. One of these effects is hematological toxicity, produced by widely used drugs such as cisplatin and characterized by leukopenia, anemia, and thrombocytopenia, leading to an increased risk of infections, bleeding disorders, and fatigue [68]. Additionally, anthracycline-derived chemotherapy drugs, such as adriamycin and epirubicin, or other agents, such as etoposide, can induce cardiomyopathy, resulting in cardiac dysfunction and a higher risk of long-term adverse cardiovascular events [69]. In addition, there is a risk of nephropathy with these drugs, since methotrexate and cisplatin can cause acute or chronic renal lesions, affect renal filtration capacity, and promote the accumulation of toxins in the body and the appearance of infections. The risk of gonadal suppression and sterility in patients treated with ifosfamide should also be noted [70]. It is imperative for patients to promptly inform their medical team of any side effects experienced, as therapeutic interventions and supportive measures are available to alleviate these symptoms and enhance quality of life during chemotherapy treatment.

The role of radiotherapy in the treatment of bone sarcomas, and specifically in OS, is limited and is also controversial, as this type of tumor is radioresistant. Therefore, radiotherapy is sometimes considered as a treatment option when surgery is not possible [71]. The most common complication for this type of tumor in the head and neck is local recurrence [38]. In our series, three cases with local recurrence were noted. Due to the anatomical complexity and the difficulty in achieving free margins, the maxilla is particularly prone to local recurrences.

OSJs do not usually metastasize and, if they do, it tends to occur in more advanced stages of the tumor, unlike in OSs in the rest of the skeleton [72]. This behavior may be related to differences in the embryonic origin of the craniofacial bones, which is derived from neural crest cells rather than hematopoietic progenitor cells. The authors of [73] indicated that the lower expression of the *GLI1* gene in craniofacial OS can be interpreted as a lower activation of the hedgehog signaling pathway. This implies a greater polarization of the M1 protein and less activation of Hh in craniofacial OS, thus explaining the low incidence of OS metastasis in this location [74]. We have not been able to corroborate these data, as there was only one case of metastasis in our series.

Regarding prognosis, it has been reported that the chondroblastic form of OS has a better prognosis than other histological types [2,32,34,38,75]. Paparella et al. [33] reported a worse prognosis in this histological variant, as they observed greater morphological variations in the nucleolar organizer regions, which act as a marker of cell proliferation and indicate a more aggressive behavior of this variant. In this regard, we did not find any differences in terms of the conventional patterns (osteoblastic, chondroblastic, and fibroblastic), but we did find differences in the epithelioid form, which was associated with a poor prognosis. There exists a consensus in recognizing that high-grade tumors [32] and those with incomplete resection or local recurrence [30] have worse prognoses. Additionally, the presence of larger lesions is associated with lower survival rates [35].

A recent study investigated possible biomarkers indicating tumor progression in terms of possible tumor recurrence and metastasis development, overall survival, and disease-free survival. It was observed that the presence of immune-environment biomarkers such as CD163 is associated with a worse prognosis in OSJ [76]. 

The lower frequency of metastases in OSJs probably influences the survival results [77,78], with 5-year survival rates of approximately 77% for craniofacial OS and 55–70% for conventional OS [58]. These data are highly variable due to the small number of cases in previous studies. In the specific cases of OSJ, the 5-year survival rate ranges from 45% to 74% [29,32,58,72,79,80]. In our study, similar results were observed, since a survival of 75% was observed within the same period. However, the limited number of cases in this study must be taken into account when interpreting the results.

A recent investigation conducted by Brown et al. examined data pertaining to the presence and impact of metastatic disease in osteosarcoma of the jaw (OSJ) within a cohort comprising 164 cases, thereby corroborating findings evident in the prior literature [35]. Despite the association between metastatic occurrences and diminished survival rates, the frequency of metastatic occurrences remains notably lower in comparison to osteosarcomas occurring in other anatomical sites. Specifically, the incidence of metastases at the time of diagnosis among patients with mandibular osteosarcoma exhibited a marked decrease (2.6%) when contrasted with those presenting with osteosarcomas affecting the upper (21.9%) and lower extremities. This diminished occurrence of metastases likely contributes to the comparatively favorable survival outcomes observed in cases of mandibular osteosarcoma in contrast to alternative presentations of the disease. Nonetheless, within their analysis, the overall survival rates of patients afflicted with mandibular osteosarcoma exhibited statistical significance only in relation to tumors localized in areas such as the pelvis and spine rather than those in the extremities [35].

Postoperative care is pivotal for patients with jaw tumors, encompassing and including speech rehabilitation, nutritional support, and psychological well-being [81]. Speech therapy is crucial for managing articulatory challenges post-surgery [82], while dietary modifications address potential malnutrition due to impaired chewing and swallowing. Psychological support is essential to address the emotional impact of surgery and cancer diagnosis [83], highlighting the importance of interdisciplinary collaboration among surgeons, speech therapists, dietitians, psychologists, and other healthcare professionals. This comprehensive approach ensures optimal outcomes and enhances patients’ overall quality of life when undergoing jaw tumor surgery [81].

### Limitations of the Study

The limited number of cases, attributable to the rare nature of these lesions, underscores the necessity of establishing a national or international database. Such a database would facilitate multicenter studies to augment the sample size, thereby fostering a comprehensive understanding of this pathology. Moreover, it would aid in the development of more standardized protocols for adjuvant or neoadjuvant chemotherapy treatments while paving the way for exploring novel avenues of research.

## 5. Conclusions

OSJs are rare lesions that differ from conventional long-bone osteosarcomas in terms of the age of onset, response to adjuvant treatment, development of metastases, and prognostic and survival data. Discrepancies still exist between the most prevalent histological forms of OSJ and those that have a better prognosis.

Surgical treatment with margins free of tumor infiltration is the most decisive factor in terms of improving the prognosis and survival of patients with OSJ. Few large case series have been reported that provide an in-depth analysis of the most controversial points in osteosarcoma management, such as the effectiveness and response to adjuvant therapy. However, additional future studies are warranted to establish more standardized protocols for therapeutic management.

## Figures and Tables

**Table 1:** Patient characteristics.

Case	Age	Sex	Medical History	Signs and Symptoms	Bone	Side
1	51	M	Pneumothorax at 26 years	-Sensation of nasal obstruction -Progressive proptosis -Diplopia	Maxilla	Left
2	35	F	None	Painful swelling	Maxilla	Left
3	56	F	Cavum cancer (T2N2M0) at 45 years. Treated with CTX and RTX	Painful swelling	Mandible (angle)	Right
4	56	F	None	Asymptomatic mass	Mandible (body and angle)	Right
5	49	F	Uterine myoma. Breast fibroadenoma	-Inferior dental nerve hypoesthesia -Painful swelling -Dental mobility of involved teeth	Mandible (body and angle)	Left
6	47	F	Maxillar cemento-ossifying fibroma at 42 years	Asymptomatic mass	Maxilla	Right
7	10	F	Retinoblastoma at 3 years. Treated with CTX and RTX. Right eye enucleation	Painful swelling	Maxilla	Right
8	31	F	Appendectomy at 21 years	-Painful swelling -Oral cavity bleeding	Mandible (body and angle)	Left

M: male; F: female; CTX: chemotherapy; RTX: radiotherapy.

**Table 2:** Pathological characteristics of tumors.

Case	Histological Morphology	Grade	Broder’s Classification	Soft-Tissue Involvement	Lymphovascular Invasion
1	Fibroblastic	High	Grade 3–4	No	No
2	Chondroblastic	High	Grade 3	No Yes (after recurrence)	No Yes (after 3rd recurrence)
3	Epithelioid	High	Grade 3	Yes	No
4	Osteoblastic	High	Grade 4	No	No
5	Osteoblastic	High	Grade 3	Yes	No
6	Chondroblastic	High	Grade 2–3	Yes	No
7	Osteoblastic	High	Grade 4	No	No
8	Epithelioid	High	Grade 4	No	Yes

**Table 3:** Treatment characteristics and outcomes.

Case	Surgical Treatment	Final Surgical Margin	Resection	Neoadjuvant Therapy	Necrosis % Post Neoadjuvant Therapy	Adjuvant Therapy	Recurrences	Metastasis	Survival and Follow-Up
1	Partial maxillectomy and eye enucleation	+	R1	No	N/A	CTX: ifosfamide-cisplatin + RT: tomotherapy 6MV (5 cycles)	No	No	FOD/15 years
						Epirubicin (3 cycles)			
2	Partial maxillectomy	1st surgery: − 2nd surgery: − 3rd surgery: n/c 4th surgery: + 5th surgery: n/c	1st Surgery: R0 2nd Surgery: R0 3rd Surgery: n/c 4th Surgery: R1 5th Surgery: n/c	CTX: cisplatin-adriamycin (1 cycle)	N/D	After 3rd surgery (pregnant in 2nd recurrence): CTX: cisplatin-adriamycin/ Methotrexate (5 cycles)	3 local recurrences	N/D	N/D
3	Hemimandibulectomy	−	R0	No	N/A	In recurrence (no surgery decided) CTX: ifosfamide	1 local recurrence	No	Death/7 months
4	Hemimandibulectomy	−	R0	No	N/A	No	No	No	FOD/15 years
5	Hemimandibulectomy	− (<1 mm)	R0	No	N/A	CTX: cisplatin-adriamycin (6 cycles)	No	No	FOD/9 years
6	Partial maxillectomy	−	R0	No	N/A	CTX: cisplatin-adriamycin (5 cycles)	No	No	FOD/7 years
7	Partial maxillectomy	−	R0	CTX: methotrexate-adriamycin-cisplatin	95%	CTX: adriamycin (1 cycle)	No	No	FOD/4 years
8	Hemimandibulectomy	+	R1	CTX: cisplatin-adriamycin (1 cycle)	15%	CTX: ifosfamide-etoposide (1 cycle)	1 local recurrence	Yes	Death/6 months

Note: +: affected margins; −: free margins; n/c: not clear; R0: complete resection with no residual tumor; R1: resection with microscopic residual tumor; N/A: not applicable; N/D: no data; CTX: chemotherapy; RTX: radiotherapy; FOD: free of disease.

## Data Availability

The data presented in this study are available on reasonable request from the corresponding author.

## Abbreviations

OS: osteosarcoma of the extremities; OSJ: osteosarcoma of the jaw, SD: standard deviation; M: male; F: female; CTX: chemotherapy; RTX: radiotherapy; ISG-GEIS-OS2 protocol: Italian Sarcoma Group–Spanish Group for Research on Sarcomas–Osteosarcomas 2; +: affected margins; -: free margins; n/c: not clear; N/A: not applicable; N/D: no data; FOD: free of disease; GLI1: zinc finger protein GLI1 Hh: hedgehog signaling pathway; AgNORs: nucleolar organizer regions.
